# Investigating impact of slash and clear vector control strategy on blackfly population and onchocerciasis transmission in a hotspot in Nigeria

**DOI:** 10.1371/journal.pntd.0014151

**Published:** 2026-04-03

**Authors:** Monsuru Adeleke, Olabanji Surakat, Bertram Nwoke, Kenneth Opara, Hayward Mafuyai, Zarat Iwalewa, Oluwadamilare Ganiu Dauda, Quadri Adeshina, Ilias Awoniyi, Murphy Nwoke, Friday Chikezie, Clement Yaro, Francisca Olamiju, Emmanuel Emukah, Chukwuemeka Makata, Olaitan Omitola, Ayodele Babalola, Benjamin Jacob

**Affiliations:** 1 Department of Animal and Environmental Biology, Osun State University, Osogbo, Nigeria; 2 Federal University of Health Sciences, Ila Orangun, Nigeria; 3 Department of Animal and Environmental Biology, Imo State University, Owerri, Nigeria; 4 Department of Animal and Environmental Biology, University of Uyo, Uyo, Nigeria; 5 Department of Zoology, University of Jos, Jos, Nigeria; 6 Department of Biochemistry, Osun State University, Osogbo, Nigeria; 7 MITOSATH, Jos, Nigeria; 8 The Carter Center (TCC), Benin, Nigeria; 9 Neglected Tropical Diseases Unit, Federal Ministry of Health, Abuja, Nigeria; 10 Nigerian Institute of Medical Research, Yaba, Lagos, Nigeria; 11 Global Health Program, University of South Florida, Tampa, Florida, United States of America; Texas Tech University, UNITED STATES OF AMERICA

## Abstract

**Background:**

The Edo–Ondo border remains a hotspot for onchocerciasis transmission and has been designated as a special intervention zone by Nigeria’s National Onchocerciasis Elimination Committee because of its persistent transmission and high blackfly biting rates. This study evaluated the effectiveness of the “slash-and-clear” vector control strategy and annual ivermectin distribution to interrupt transmission of *Onchocerca volvulus* in the area.

**Methods:**

This study was implemented in two phases. Phase 1 (July–August 2023) involved slash-and-clear in four communities along the Ose River and its tributaries, while four others served as controls. Baseline biting rates were recorded for eight days, after which interventions were implemented in designated sites and fly collections continued for 29 days. Phase 2 (September 2023–June 2025) annual slash-and-clear was extended to 76 communities, including the original eight, where fly populations were continuously monitored. All collected blackflies were screened for *O. volvulus* infection using Ov ND5 qPCR pool screening.

**Results:**

In Phase 1, biting rates in intervention communities decreased significantly by 51.1–91.9% (p < 0.05) along tributaries but increased in communities located directly on the Ose River or within 5 km. No significant changes were observed at the control sites. In Phase 2, sustained slash-and-clear across 76 communities resulted in significant reductions in blackfly densities, ranging from 39.7%–94.5%. Analysis of the flies showed infectivity for Ov ND5 genes in designated communities in the main Ose River and its tributaries indicating the presence of reservoirs of human *Onchocerca volvulus* microfilariae of onchocerciasis in the area.

**Conclusion:**

The results of this study demonstrate the possibility of significant reduction in *Simulium damnosum* complex biting rates with sustained slash-and-clear activity in the region (most especially the tributaries). Therefore, we recommend further studies to determine drivers of transmission aside from flies to fast-track the elimination of onchocerciasis in the area.

## Author summary

Despite years of ivermectin distribution, onchocerciasis transmission persists in communities along the Ose River along the Edo–Ondo border. This region was recently designated as a special intervention zone (hotspot) by the National Onchocerciasis Elimination Committee (NOEC) of Nigeria due to persistent transmission of onchocerciasis despite the introduction of twice-yearly ivermectin distribution in 2021 to curb ongoing transmission**.** The continued transmission highlights the need to complement mass drug administration (MDA) with vector control strategies. Slash-and-clear have emerged as a promising intervention that can be integrated with MDA. The findings of this study suggest that the slash-and-clear intervention may reduce blackfly populations and, if implemented consistently and sustainably, could contribute to transmission interruption in the region. However, detection of infective flies in both the Edo and Ondo parts of the region indicates presence of reservoirs of human *Onchocerca volvulus* microfilariae of onchocerciasis in the area. and validates the designation of this zone for special intervention by the NOEC. Continued twice-yearly distribution of ivermectin, complemented by sustained implementation of slash-and-clear in communities along the long stretch of the Ose River, is therefore essential to reduce blackfly populations and accelerate the elimination of onchocerciasis in the region.

## Introduction

Human onchocerciasis is a debilitating neglected tropical disease caused by the filarial nematode *Onchocerca volvulus* and transmitted through the bite of *Simulium* vectors [1]. In West Africa, the cytospecies of *Simulium damnosum* complex are the major vector that breed profusely in fast-flowing rivers, streams, dams and spill-ways [2,3]. Onchocerciasis is endemic in 37 countries across four continents but sub-Saharan Africa accounts for about 99% of the world’s cases [4]. Of these 99% of cases in Africa, Nigeria is the most endemic contributing to approximately 40% of the global prevalence. Approximately 50 million Nigerians living in over 36,000 communities spread across 32 out of 36 sates in Nigeria and the Federal Capital Territory (FCT) is estimated to be at risk of infection [5].

The thrust of disease control (and now elimination) relies on annual chemotherapy through the use of ivermectin in endemic communities [6]. This was achieved through the community directed distributors (CDTI). Evidence indicates the potential for long-term use (at least 15 years) of the drug to eliminate onchocerciasis in endemic communities [7]. Compared with other endemic countries that have achieved onchocerciasis elimination or interruption of transmission, Nigeria has made uneven but substantial progress. Two states have achieved elimination of transmission of the disease (Plateau and Nasarawa), confirmed interruption in nine states, and is suspected to have been interrupted in eighteen states including the Federal Capital Territory. The remaining states are at varying stages of transmission interruption [5]. Despite the twice-yearly distribution of ivermectin and high therapeutic coverage achieved in communities along the Edo–Ondo border, onchocerciasis transmission has persisted, prompting the National Onchocerciasis Elimination Committee (NOEC) of the Federal Ministry of Health to designate the area as a special intervention hotspot. The ongoing transmission is driven by multiple factors, including high blackfly density, which increases human–vector contact and consequently elevates the risk of disease transmission. Consequently, to strengthen control efforts, the National Onchocerciasis Elimination Committee (NOEC) in May 2022 endorsed the programmatic use of vector control using the slash-and-clear strategy to complement the ongoing twice-yearly ivermectin distribution initiated in 2021, with the programmatic objective of reducing blackfly biting rates and supporting progress toward elimination in the region [8].

The “slash-and-clear” strategy have been successfully used as vector control strategy in Uganda, Cameroon and Sudan [9–12]. The slash-and-clear intervention is a community-directed vector control technique that entails the identification and physical slashing and removal of vegetations in blackfly breeding sites using machetes. The underlying premise is that clearing these vegetations eliminate attachment surfaces for blackfly eggs, causing them to be washed away by water flow. This disruption of early developmental stages leads to a reduction in blackfly populations and, consequently, lower biting rates and reduction in transmission risk *of O. volvulus***.** The effectiveness of the slash-and-clear method in reducing blackfly populations has been demonstrated in hypo-endemic settings and communities with small, easily accessible rivers [9,10,12]; however, the impact of this intervention in several hyper-endemic communities characterized by large rivers and high vector abundance remains unproven. It is against this background that this study was conducted to evaluate the effectiveness of the slash-and-clear intervention as a vector control strategy in multiple hyper-endemic communities along the Ose River and its tributaries on the Ondo–Edo border, and to assess the effectiveness of twice-yearly ivermectin distribution in interrupting onchocerciasis transmission in selected communities, with blackfly infectivity used as indicator of transmission status in the region.

## Materials and methods

### Ethics statement

The study protocol was approved by the National Health Research Ethical Committee of the Federal Ministry of Health, Nigeria (NHREC/01/01/2007-13/03/2023). The Federal Ministry of Health (NTDs Units), the State Coordinators of the NTDs and the community leaders in the study communities were mobilized for the study. The team obtained written informed consent from the community leaders and also from each of the Human Landing Collectors (HLCs) that participated in the study after the objectives, benefits and risks had been explained and understood by them. All the HLCs and those recruited for the slash-and-clear intervention were also administered ivermectin prior the study and after the study. Human landing catch (HLC) collections were conducted in accordance with the protocol approved by the Ethics Review Committee of the Federal Ministry of Health and in line with international ethical guidelines.

### Description of study area

**Fig 1:**
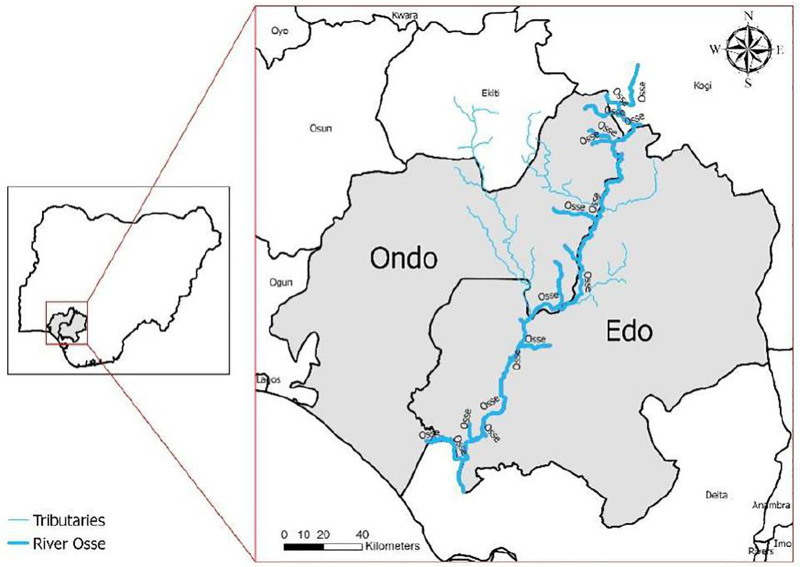
Map of Edo-Ondo border with the river system.

The study was carried out in communities around the Ose River along the Ondo-Edo states border (Fig 1). Ose River is a large, forested river that runs along the border of Edo and Ondo States and has several tributaries in both Edo and Ondo states which empty their content into the Ose River. The river falls approximately within the coordinates 5⁰ 50‵ - 7⁰ 50‵ N and 5⁰ 0‵ - 6⁰ 10‵ E in Southern Nigeria [13]. The river and its tributaries contain Precambrian rocks and vegetation that are scattered at different intervals. Typha and Andropogon species were the dominant grasses along the water course while *Pterocarpus santalinoides* was the predominant tree.

The administrative boundary shapefile was obtained from geoBoundaries: a global database of political administrative boundaries available at https://www.geoboundaries.org. The Ose River system shapefile was obtained from AQUASTAT (FAO) Rivers of Africa dataset available at https://data.apps.fao.org/catalog/iso/b891ca64-4cd4-4efd-a7ca-b386e98d52e8. Both datasets are provided under the Creative Commons Attribution 4.0 International (CC BY 4.0) license.

### Study design

(a) Phase 1: Implementation of the “slash-and-clear” along the Edo-Ondo border

This phase was conducted between July 3 2023 and August 4, 2023. Eight communities were selected along the Ondo and Edo states (Table 1) for the preliminary study on slash-and-clear. The eight communities were purposely selected to represent first-line communities along the Ose River and its tributaries and to ensure the safety of the team in accessing the community. Larval prospection was conducted by trained entomologists to confirm the active breeding sites of *Simulium damnosum s.l* in selected communities. Human landing collectors (HLCs) were then recruited and trained at each location. Fly collection was conducted in all eight communities for consecutive 8 days using the standard HLC method [14]. The HLCs consisted of two young men per community working alternately on an hourly basis between 07:00 and 18:00hr to obtain baseline blackfly biting data following the procedure of Raimon *et al* [10]. Four of the communities were designated as intervention communities and the remaining were designated as control villages. The human landing collection of the flies were sustained in all eight communities until day 27. The data collected on biting population dynamics were analyzed. Fly collection was subsequently conducted regularly for two consecutive days per month. All flies collected from each location were preserved in 80% ethanol for identification and molecular analysis.

**Table 1:** The description of the selected locations for monitoring of fly population pre and post slash-and-clear.

S/N	State	Name of community	Latitude	Longitude	River type	Distance to the main river (km)	Designation
1	Edo	Ikhin	7.14178416	5.95326	Tributary	6.65	Intervention
2	Edo	Uroe	7.11263151	5.94831	Tributary	9.18	Control
3	Edo	Okpokhumi	6.94217343	5.99294	Tributary	20.63	Intervention
4	Edo	Atoruru	6.85001971	5.86239	Tributary	6.43	Control
5	Ondo	Idogun Community	7.30694	5.922357	Main river	0	Intervention
6	Ondo	Idogun bypass	7.322878	5.920582	Main river	0	Control
7	Ondo	Sinabole-Owani	7.239357	5.804286	Tributary	12.8	Intervention
8	Ondo	Olorunsogo	7.233053	5.819442	Tributary	8.11	Control

### The slash-and clear intervention strategy

The slash-and-clear were conducted following procedure of Jacob *et al* [9] on days 9–12 in the intervention communities only. Following baseline blackfly collections, six young men (aged 18–40 years) were carefully recruited from each intervention community based on their familiarity with the river terrain and swimming ability. The recruits were taken to identified blackfly breeding sites located upstream and downstream of the communities and trained to slash and remove trailing and submerged vegetation from the river. The removed vegetation was discarded on riverbanks to dry, thereby destroying attached larvae and pupae. Slash-and-clear activities covered a 1 km stretch upstream and downstream of each intervention site.

(b) Phases 2 (2024) and 3 (2025) of slash-and-clear along Edo-Ondo border

A total of 79 communities were identified along the Ose River and its tributaries, including four intervention sites and four selected control sites in Phase 1. The community leaders and stakeholders were well mobilized in all communities with the help of the State NTDs teams and Local Governments NTDs teams. Slash-and-clear were conducted in 71 communities in 2024 before the commencement of the wet season (April - May, 2024). Three communities declined or were not reached (due to insecurity and communal clashes) and one community declined to participate attributing the slash-and-clear as a destructive way to the livelihood of the residents. The community is a fishing community and claimed that the vegetation serves as source of food for the fish in the river upon which the socio-economic fortune of the residents depends. Despite all efforts to disabuse their minds through social engagement before and during the study, it appeared to have little or no impact on their determination not to participate in slash-and-clear. Unexpectedly, the four communities designated as control communities in phase 1 independently carried out slash-and-clear after observing neighbouring communities undertaking the intervention, despite our team’s prior explanation to residents that their communities were designated as control. Their actions were influenced by a circulating report suggesting that slash-and-clear reduces blackfly populations locally and that participating communities will continue to benefit from government support. This altered our study design of having control beyond phase 1. This was a significant limitation of the study.

(c) Phase 3 was a repeat of the slash-and-clear in 2025 in all the communities that participated in Phase two including the four control communities. The fly population was monitored in all eight communities throughout the study, as explained earlier in Phase 1. All *S. damnosum* flies collected were preserved as explained earlier in Phase 1.
(d) qPCR analysis of the fly heads for infectivity

The flies collected on a daily basis between July and August 2023 and those collected between September 2023 and May 2024 were analyzed using the Ov ND5 genes protocol as detailed in Hendy *et al.,* [15]. The flies collected between June 2024 and June 2025 were analyzed using an improved procedure described by Smith College on 0–150-Ov ND5 procedure (see S1 File for detailed procedure). Data on the distribution of ivermectin in the communities were also obtained from the eight selected communities.

### Data analysis

Data was collected from two states and analyzed separately by state using an identical analytical framework. All analyses were conducted using negative binomial mixed-effects models to evaluate changes in blackfly counts across study periods following slash-and-clear interventions. Blackfly counts were modelled using a negative binomial mixed-effects model with a log link (nbinom1). For each state-specific analysis, study period and site were included as fixed effects, and a random intercept was specified for the sampling unit (collection period) to account for repeated measurements over time. Alternative negative binomial parameterizations (nbinom2, nbinom12) were evaluated during model development. Model comparison based on information criteria consistently favoured the nbinom1 specification, which showed lower AIC, BIC, and deviance values. Likelihood ratio tests confirmed statistically significant improvements in model fit, and the nbinom1 model was therefore retained for all final analyses. Potential interactions between study period and site were examined to evaluate site-specific intervention effects. Although including the interaction term increased multicollinearity, it was retained because it significantly improved model fit (p < 0.05, Likelihood Ratio Test) and reflected biologically plausible variation in treatment efficacy across sites. In contrast, models with only main effects exhibited negligible collinearity. Based on these results, interaction terms were maintained in the final model. The model was also compared with a version including no random effects, where site, study period, and collection time were modeled solely as fixed effects, including the site × study period interaction. The model incorporating random effects demonstrated superior fit, supporting its retention. Estimates from the final model were expressed as Incidence Rate Ratios (IRRs) by exponentiating the original coefficients. Predicted means were generated on the original count scale using estimated marginal means, (*emmeans,* type = “response”). Comparisons between post-intervention periods and baseline (Before slash and clear) were expressed as ratios (After / Before), and percentage reduction was calculated as (1 − ratio) × 100. Statistical significance was assessed at a two-sided α level of 0.05. Across both state-specific analyses, model diagnostics indicated adequate overall fit. No strong evidence of overdispersion was detected. Model explanatory power was high, with marginal R² values indicating substantial variance explained by fixed effects and conditional R² values demonstrating a strong contribution from random effects. Adjusted intraclass correlation coefficients (ICC) indicated moderate to strong clustering within sampling units. All analyses were conducted in R (version 4.4.3) using RStudio (version 2026.01.0 + 392). Mixed-effects models were fitted using the *glmmTMB* package (version 1.1.13). Estimated marginal means were obtained using *emmeans* (version 2.0.1). Model diagnostics were performed using the *performance* package (version 0.15.3).

## Results

a Results of slash-and-clear intervention

**Fig 2:**
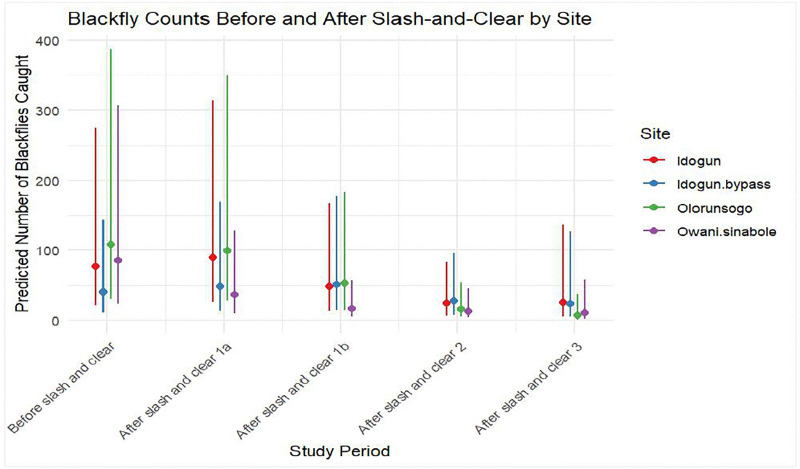
The population dynamics of the blackflies pre and post intervention during the study at Ondo border.

**Fig 3:**
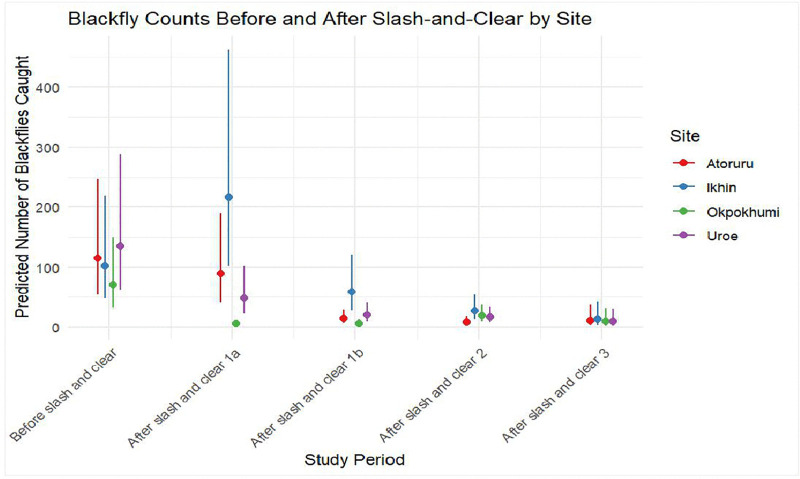
The population dynamics of the blackflies pre and post intervention during the study at Edo border.

The results from Phase 1 showed that the biting rates were reduced by 51.1% after the slash-and-clear in Sinabole-Owani, an intervention community/site on a tributary to the Ose River. This reduction was statistically significant (p = 0.026)**.** However**,** the biting rate increased (16.1%) at the Idogun community (intervention) located on Ose River after slash-and-clear but the difference was not statistically significant (p = 0.939). There was no significant change in biting rates at both control sites (without intervention) in the pre and post intervention period in the communities located at the Ondo State border (Fig 2 and Tables 2 and 3). On the Edo State side of the border, the biting rates reduced significantly (p = 0.001) by 92% after slash-and-clear in the Opokhumi (intervention), a tributary to the Ose River. The biting rate, however, increased significantly (p = 0.015) by 112% at Ikhin, a tributary about 6km from the Ose River after slash and clear. No significant change (p = 0.684) in biting rate was observed at Atoruru (a tributary), a control site in the pre and post intervention period but a significant reduction was observed at Uroe (a tributary to Ose River), a control village in post intervention period (Fig 3 and Tables 4 and 5).

**Table 2:** Mean blackfly counts (95% CI) before and after slash-and-clear by site and phase at Ondo.

Site	Mean count (95% CI) Before slash and clear	Mean count (95% CI) After Phase 1 (Daily; July-August, 2023)	Mean count (95% CI) After Phase 1 (6 months)	Mean count (95% CI) After Phase 2(2nd year; 16 months)	Mean count (95% CI) After Phase 3(3rd year; 24 months)
Idogun community	61.0 (17.1–218.4)	70.9 (20.2–249.5)	38.5 (11.2–132.6)	19.4 (5.7–66.3)	20.8 (4.0–108.3)
Idogun Bypass	32.0 (8.9–114.5)	38.2 (10.9–134.5)	40.9 (11.9–140.7)	22.4 (6.6–76.5)	19.3 (3.7–100.6)
Olorunsogo	86.0 (24.0–307.7)	79.1 (22.5–278.2)	42.2 (12.3–145.4)	12.7 (3.7–43.4)	5.6 (1.0–30.0)
Owani sinabole	68.0 (19.0–243.5)	28.9 (8.2–101.9)	13.1 (3.8–45.6)	10.4 (3.0–35.7)	8.8 (1.7–46.5)

**Table 3:** Percentage reduction of blackfly counts after slash-and-clear relative to baseline by site and phase at Ondo.

Site	Period	Percent reduction	p-value	Evidence
Idogun	After Phase 1(Daily; July-August, 2023)	−16% (increase)	0.939	Not significant
Idogun	After Phase 1(6 months)	37%	0.373	Not significant
Idogun	After Phase 2(2nd year; 16 months)	68%	<0.001	Strong
Idogun	After Phase 3(3rd year; 24 months)	66%	0.261	Not significant
Idogun Bypass	After Phase 1(Daily; July-August, 2023)	−20% (increase)	0.911	Not significant
Idogun Bypass	After Phase 1(6 months)	−28% (increase)	0.806	Not significant
Idogun Bypass	After Phase 2 (2nd year; 16 months)	30%	0.565	Not significant
Idogun Bypass	After Phase 3 (3rd year; 24 months)	40%	0.801	Not significant
Olorunsogo	After Phase 1(Daily; July-August, 2023)	8%	0.985	Not significant
Olorunsogo	After Phase 1(6 months)	51%	0.078	Weak
Olorunsogo	After Phase 2 (2nd year; 16 months)	85%	<0.001	Very strong
Olorunsogo	After Phase 3 (3rd year; 24 months)	94%	<0.001	Very strong
Owani Sinabole	After Phase 1(Daily; July-August, 2023)	57%	0.026	Strong
Owani Sinabole	After Phase 1(6 months)	81%	<0.001	Very strong
Owani Sinabole	After Phase 2 (2nd year; 16 months)	85%	<0.001	Very strong
Owani Sinabole	After Phase 3 (3rd year; 24 months)	87%	0.005	Strong

*P* value greater than 0.05 is significant using generalised linear mixed model.

**Table 4:** Mean blackfly counts (95% CI) before and after slash-and-clear by site and phase at Edo.

Site	Mean count (95% CI) Before slash and clear	Mean count (95% CI) After Phase 1 (Daily; July-August, 2023)	Mean count (95% CI) After Phase 1 (6 months)	Mean count (95% CI) After Phase 2(2nd year; 16 months)	Mean count (95% CI) After Phase 3(3rd year; 24 months)
Atoruru	96.6 (45.0–207.2)	74.3 (34.8–158.5)	11.8 (5.6–24.7)	7.2 (3.5–14.8)	9.4 (2.8–31.3)
Ikhin	85.6 (39.9–183.7)	181.7 (85.2–387.1)	48.6 (23.4–101.1)	22.3 (10.9–45.5)	10.6 (3.2–35.3)
Okpokhumi	58.5 (27.3–125.7)	4.7 (2.2–10.3)	5.1 (2.4–11.1)	15.6 (7.6–31.9)	7.8 (2.3–26.4)
Uroe	112.5 (52.5–241.5)	40.1 (18.8–85.7)	16.5 (7.9–34.4)	14.2 (6.9–29.0)	7.5 (2.2–25.4)

**Table 5:** Percentage reduction of blackfly counts after slash-and-clear relative to baseline by site and phase.

Site	Period	Percent reduction	p-value	Evidence
Atoruru	After Phase 1 (Daily; July-August, 2023)	23%	0.684	Not significant
Atoruru	After Phase 1 (6 months)	88%	<0.001	Very strong
Atoruru	After Phase 2 (2nd year; 16 months)	93%	<0.001	Very strong
Atoruru	After Phase 3 (3rd year; 24 months)	90%	<0.001	Very strong
Ikhin	After Phase 1 (Daily; July-August, 2023)	−112% (increase)	0.015	Evidence of increase
Ikhin	After Phase 1 (6 months)	43%	0.087	Weak
Ikhin	After Phase 2 (2nd year; 16 months)	74%	<0.001	Very strong
Ikhin	After Phase 3 (3rd year; 24 months)	88%	<0.001	Strong
Okpokhumi	After Phase 1 (Daily; July-August, 2023)	92%	<0.001	Very strong
Okpokhumi	After Phase 1 (6 months)	91%	<0.001	Very strong
Okpokhumi	After Phase 2 (2nd year; 16 months)	73%	<0.001	Very strong
Okpokhumi	After Phase 3 (3rd year; 24 months)	87%	0.001	Strong
Uroe	After Phase 1 (Daily; July-August, 2023)	64%	<0.001	Strong
Uroe	After Phase 1(6 months)	85%	<0.001	Very strong
Uroe	After Phase 2 (2nd year; 16 months)	87%	<0.001	Very strong
Uroe	After Phase 3 (3rd year; 24 months)	93%	<0.001	Very strong

*P* value greater than 0.05 is significant using generalised linear mixed model.

### Phase 2 and 3

The results of the phase 2 and phase 3 analysis showed that there was a significant reduction in the fly population across all eight fly monitoring locations. This reduction ranged from 39.66–94.49% (Figs 2 and 3 and Tables 2–5). Meanwhile, we observed breeding (larvae) of *S. damnosum* s.l on some Precambrian rocks at the Ose River during the study.

The seasonal random effect variance was estimated as 0.85 (Standard Deviation = 0.85), confirming substantial differences between the two collection seasons. Ignoring season would have overstated precision, so the mixed model approach was necessary. The faceted visualizations reveal that seasonal changes play a critical role. The blackfly abundance was higher during the wet season and crashed to near-zero during the dry season.

b) Blackfly infectivity

The results of the pool screening analysis of the fly head conducted during phase 1 of slash-and-clear (July - August, 2023) showed that none of the pools were positive for Ov ND5 genes in the selected four communities on each of Ondo and Edo sides of the border. However, the analysis of the flies (September 2023 -May, 2024) showed infectivity for *O. volvulus* ND5 genes in Idogun bypass, Idogun community and Olorunsogo on the Ondo side of the border. No positive pools were detected in Owani. Similarly, the pool analyzed in 2025 (June 2024 to June 2025) using an improved method showed confirmed cases of infectivity for 0–150 and Ov ND5 genes in Idogun bypass, Idogun community and Olorunsogo, whereas cases of positivity for either O-150 or Ov ND5 genes were observed at Owani (Sinabole), Idogun bypass and Idogun community in Ondo State which were scored inconclusive. On the Edo side of the border, analysis of flies collected between September 2023 and May 2024 revealed *O. volvulus* ND5 gene infectivity in Ikhin and Uroe, while no pools of fly heads from Okpokhumi and Atoruru tested positive. In 2025, pools positive for O-150 ND5 genes were detected in Ikhin, Uroe, and Okpokhumi. A case of positivity for either O-150 or Ov ND5 genes was also observed in Ikhin but was classified as inconclusive. None of the pools from Atoruru were positive for either Ov ND5 or O-150 gene (Table 6). When the data for each state border was pooled, the infectivity was 0.124 in Ondo while Edo hd infectivity rate of 0,060. The upper bound interval of prevalence (95% CI) was 0.187 and 0.110 for Ondo and Edo States respectively (Table 7).

**Table 6:** Pool screening Analysis of the fly heads using Ov ND5 genes.

Locations	No of flies screened	No of pools screened	No positive	No inconclusive
Ondo Communities, July - August 2023				
Idogun bridge	2232	23	0	0
Idogun community	4189	42	0	0
Olorunsogo	3173	52	0	0
Owani	2916	30	0	0
Edo Communities, July - August 2023				
Ikhin	5842	59	0	0
Uroe	3236	33	0	0
Okpokhumi	1322	14	0	0
Atoruru	3668	37	0	0
Ondo Communities, September 2023 - May 2024				
Idogun bridge	645	7	7	0
Idogun community	472	5	5	0
Olorunsogo	498	5	2	0
Owani	197	2	0	0
Edo Communities, September 2023 - May 2024				
Ikhin	552	6	2	0
Uroe	189	2	1	0
Okpokhumi	144	2	0	0
Atoruru	227	3	0	0
Ondo Communities, June 2024 - June 2025				
Idogun bridge	798	8	5	1
Idogun community	623	7	2	1
Olorunsogo	294	3	1	0
Owani	394	4	0	1
Edo Communities, June 2024 - June 2025				
Okpokumi	346	4	3	0
Uroe	242	3	1	0
Atoruru	189	2	0	0
Ikhin	589	6	3	1

**Table 7:** The infectivity and (95% CI) of the blackfly heads by state.

States	No of heads pool screened	No of pools screened	No of pools positive	Infectivity	Lower and Upper (95% CI)
Ondo	16, 431	188	22	0.124	0.0817– 0.187
Edo	16, 546	171	10	0.060	0.0325 – 0.110

c) **Observations on ivermectin distribution in the selected 8 communities**

Ivermectin was distributed once a year (April/May) in 2023 and 2024 in communities on the Ondo side of the border. The drug was not distributed as of June, 2025. In Edo State, Ivermectin treatment was conducted twice in 2023 and 2024 (April and November, 2024) and May, 2025.

## Discussion

The border area between the Ondo and Edo states is one of the hotspot foci of onchocerciasis in Nigeria. The focus was declared as a special intervention zone due to the suspected high biting of the fly and continued transmission of onchocerciasis [8]. Our baseline assessment (phase 1) showed intense breeding and biting of blackfly vectors in the area. The Ose River and its tributaries that transverse the communities around the border of both states are ecologically suitable to support the localized breeding of blackflies. The Ose River and its tributaries are fast-flowing with forested vegetation and Precambrian rocks which create rapids and vegetational support for *Simulium damnosum* complex [16–17]. These optimum ecological conditions would have resulted in a high biting density of blackflies in the area and continued human-vector contact. A previous study by Ajayi & Olusi [18] on blackfly distribution in Ondo State also reported the breeding of *Simulium* vectors along the Ose River.

Observations from the Phase 1 trial suggest that slash-and-clear may have contributed to a reduction in fly biting rates in tributaries located at least 15 km from the main Ose River, though no noticeable effect was observed on the main river or on tributaries within 5 km of the river, such as Ikhin. The Ose River is a large river with active breeding sites running through its stretch, especially during the wet season. Our current study revealed that it is difficult to conduct effective slash-and-clear in the Ose River or any of its close tributaries once the rainy season has taken full discharge. The worsening scenario is the observed breeding of flies on rocks that are immovable and could not be removed during the slash-and-clear. These sites may have provided sustained breeding and larval support, thereby limiting the effectiveness of slash-and-clear along the main river. Similar observations were reported by Lakwo et al [12] in some rivers in southern Sudan. The ineffectiveness of the slash-and-clear in Ikhin (5 km away from the Ose River) may be attributed to its proximity to Ose River, as the flies biting at this location are presumed to be a mixed population of flies coming from the tributary and the main Ose River as blackflies can move over 20 km away from the breeding sites [12].

Interestingly, we observed a significant reduction in the fly population across all the eight fly monitoring sites (including communities in the main Ose River) when the slash-and-clear were conducted in over 70 communities across the border of both states. The slash-and-clear in phases 2 and 3 were conducted at the onset of the wet season in all the communities, which could have destroyed the potential larval habitats and made it difficult for breeding sites colonization to occur at the peak of the wet season in the area including the main Ose River, as the biting rate of blackflies has been reported to naturally crash in the dry season because of the absence of rainfall and free flow of rivers in Southern Nigeria [19–20]. This approach has been described as optimum and perhaps reinforced the importance of timing in the conduct of slash-and-clear for effective outcomes as previously posited by Jacob et al [9]. Even though the larvae of the fly were recovered from rocks, this habitat has been described as a poor larval support in the absence of vegetation [12], thus leading to low fly population for two consecutive years (2024 and 2025). The potential of rocks harbouring larvae has implications as it offers sustained habitat for the flies and could expedite population recovery if slash-and-clear interventions are interrupted.

We observed enthusiasm among the residents of the study area to participate in vector control efforts, but we assumed that this enthusiasm was remotely driven by anticipated government intervention. Nevertheless, our interaction showed that the community members will be willing to conduct this vector control activity with little incentives, as is the case for community distributors of ivermectin. This strategy could sustain slash-and-clear approach as community-driven vector control strategy. Despite the perceived positive impact of the slash and clear in the area, there were challenges, including insecurity, the forested nature of the river, and demand for incentives as the participants are deemed to abandon their farms for several days (due to the forested nature of the Ose River and its tributaries).

The results from the pool screening analysis of fly heads suggest the presence of reservoirs of human *Onchocerca volvulus* microfilariae of onchocerciasis in the study area. Sustained biannual distribution of ivermectin is expected to accelerate suppression of transmission in hyperendemic areas by keeping microfilarial loads very low, thereby reducing the likelihood of uptake by blackflies during blood feeding on human hosts [7]. The zero positivity in flies screened between July and August, 2023 may be attributed to the effect of ivermectin on microfilaria load as the drug was distributed in all the communities shortly before the commencement of our study. Ivermectin is a microfilaricide known to kill microfilariae that can be ingested by flies feeding on infected human hosts [6].

The utilization of the novel qPCR method to detect the DNA of *O. volvulus* is an added advantage. The technique is more sensitive than 0–150 PCR-ELISA as recently reported by Doherty et al [21] and Adeleke et al [14]. The modified protocol recently developed by Smith College which combines both 0–150 genes and Ov NDs genes has addressed most of the initial issues associated with the Ov ND5 technique and its acceptability. This includes the addition of a non-interfering *Bacillus* Universal extractor to monitor inhibition and contamination and simultaneous monitoring of both 0–150 genes and Ov NDs genes in the same assay. The inconclusive pools are pools that are either positive for any of the targeted genes, and sequencing would be required for a definitive decision to determine if the identified gene is *O. volvulus*. This is a second limitation but will be resolved in future studies.

## Conclusion

The impact of S&C was hardly noticeable at the small scale (Phase 1), but the effect was more evident when the intervention was extended to more communities in the area (Phase 2 and 3). The results, therefore suggest that, when implemented under appropriate ecological and operational conditions, sustained slash-and-clear across the communities along the Edo-Ondo border may reduce biting rates and, when integrated with mass drug administration (MDA), could ultimately contribute to transmission interruption along the Edo-Ondo border. However, persistent breeding on rock substrates may facilitate blackfly recolonization over time, particularly along the Ose River. The detection of *O*. vol*vulus DNA* in the flies suggest presence of human reservoir of the parasite in the study area. Therefore, we recommend further studies to understand the drivers of the continued onchocerciasis transmission in the study area.

## Supporting information

S1 File Improved procedure described by Smith College on 0–150-Ov ND5. (DOCX)
