# Supplementary material for: Investigating impact of slash and clear vector control strategy on blackfly population and onchocerciasis transmission in a hotspot in Nigeria
Source: PLoS Negl Trop Dis. 2026 Apr 3;20(4):e0014151. doi: 10.1371/journal.pntd.0014151 (PMC13048400; doi:10.1371/journal.pntd.0014151)
Supplement: S1 File — (DOCX) [file pntd.0014151.s001.docx]

**PROTOCOL FOR O-volvulus GENE DETECTION USING qPCR**

(Adapted from the improved protocol developed by Prof Steven Williams, Smith College, United State of America*)*

1. **Separation of heads from body**

1. Documentation of each *Simulium* blackfly pool in a separation record sheet.

2. Briefly, centrifuge the tube containg the pool of blackflies in a 1.5/2.0mL tube

- 1. 3. Rinse the blackflies twice in 95% ethanol

4. Add an additional volume of 95% ethanol (1-2 mL) to the tube with flies and pour everything out onto a plastic weigh boat. Rinse the remaining flies from the tube with a transfer pipette.

- 1. 5. Count the number of flies in the pool to ensure accuracy of field collected data and record on the blackfly head separation record sheet.
  2. 6. Use a transfer pipette (wide bore) to transfer the ethanol and flies into a clean, dry and labeled 15 mL polypropylene conical tube.
  3. 7. Add additional ethanol to the weigh boat to help collect the flies into the transfer pipette.

8. Remove the excess ethanol from 15mL conical tube using a transfer pipette. then, use a P200 pipette tip to remove any remaining ethanol from the flies.

8. Gently spread the blackflies around the side of the 15mL conical tube and allow the ethanol to evaporate for 10-15 minutes until the flies are mostly dry. Note, Do not allow the flies to desiccate completely to avoid unsuccessful head separation.

9. Place the 15mL conical tubes at –80oC overnight or in the vapour phase of liquid nitrogen for 30 min.

10. Remove the frozen 15-mL conical tube containing the frozen blackflies

- 1. 11. Immediately start tapping the tube on a laid foam pad vigorously against the laboratory bench top.

1. Resuspend the separated heads and bodies in 95% ethanol and transfer them from the tube to a large plastic weigh boat with a transfer pipet.
2. Inspect the flies under a dissecting microscope ≥ 80-90% of the heads should be separated if procedure was performed properly.
3. Collect the heads from the weigh boat using a transfer pipette and transfer them into a separate weigh boat. Count the number of heads – the number of heads should match the number of flies in the original pool size.
4. Once all the heads are accounted for, transfer the ethanol and heads to a sterile 2.0 mL round bottom tube, labeled as follows: HXXX, where H=Head, and XXX is the pool sample number. The tube should also be labeled with the date and your initials.
5. Transfer the bodies using a wide bore transfer pipette to different sterile 2.0 mL round bottom tube and label as follows: BXXX, where B=Body and XXX is the pool sample number.
6. Add additional 95% ethanol if needed to ensure the bodies are submerged. The tube should also be labeled with the date and your initials.
7. Record the number of heads and bodies recovered on the blackfly head separation record sheet. Record any comments/notes from the separation procedure on this sheet.
8. Place the tubes in a labeled box in the 4oC refrigerator. Keep the samples in a 4oC fridge until the DNA extraction step.

**ALTERNATIVELY**

The head can be separated manually using dissecting pin to separate the heads from the bodies under the dissecting microscope as described by Hendy *et al.,* (2018)

1. **NUCLIEC ACID EXTRACTION (QIAGEN DNeasy Blood & Tissue Kits (Qiagen, N.V.)**

**Notes before starting:**

- Perform all centrifugation steps at RT
- Redissolve any precipitations in Buffer AL and Buffer ATL
- Add ethanol to Buffer AW1 & AW2 concentrates
- Equilibrate frozen tissue or cell plates to RT
- Preheat an incubator to 56℃

1) Prepare a negative extraction control by labeling a 2mL round bottom microcentrifuge tube as negative extraction control or “ExC”.

2) Transfer each pool of blackfly heads to a 2mL round bottom microcentrifuge tube if they are not already stored in this type of tube.

3) Remove all of the ethanol from each 2mL round bottom microcentrifuge tube with blackfly heads using a 200uL and/or 20uL pipette. Make sure not to lose any heads in the process.

4) Leave the 2mL round bottom microcentrifuge tube cap open, covering with a kimwipe, to allow the ethanol to evaporate off the blackfly heads.

5) Add 1 stainless steel bead to each 2mL round bottom microcentrifuge tube containing up to 100 black fly heads. **Do not add a stainless-steel bead to the negative extraction control.**

6) Add 170μL 1X PBS to each sample including the negative extraction control.

7) Add 10μL of stock extraction control, *B. atrophaeus,* to each sample with blackflies and to the negative extraction control.

8) Place tubes with blackflies in the TissueLyser II/III aluminum blocks. **Do not add the negative extraction control tube to the TissueLyser II/III aluminum blocks, keep on the bench while the blackfly pool samples are macerated.** Ensure tubes are labeled on the sides of the tube. The TissueLyser II processing will remove labeling on the top of the tubes.

9. Macerate samples for 10 minutes at a frequency setting of 30.0 Hz.

10) Centrifuge samples briefly to collect material away from the lid of the tube.

**11)** Process the negative extraction control the same as the other samples**.** Add 20 μL of Qiagen Proteinase K to each sample tube and the negative extraction control.

12) Add 200 μL of Buffer AL to each sample tube. Immediately mix each sample by vortexing the tube long enough to resuspend any pelleted material (about 3 seconds)

13) Incubate samples at 70°C for 10 minutes in a TempBlock incubator.

14) Centrifuge tubes briefly to collect material away from the lid of the tube.

15) Add an additional 20 μL of Qiagen Proteinase K to each sample. Mix all samples by vortexing the tubes just long enough to resuspend any pelleted material (about 3 seconds).

16) Incubate all tubes at 56°C for 1 hour in a TempBlock incubator.

17) Centrifuge all tubes at maximum speed for 5 minutes to pellet debris.

18) While samples are centrifuging, label 1.5 mL microfuge tubes and add 200 μL of 95% ethanol to each.

19) Label DNeasy spin columns. Prepare one spin column for each sample

20) Transfer the supernatant from each sample tube to a 95% ethanol-containing tube prepared in step 16. Using the same pipette tip that was used to transfer each sample, gently pipet the sample-ethanol mixture up and down to mix, then apply the entire sample to a DNeasy spin column. (Note: Be sure to use a microcentrifuge tube opener to open all sample tubes.).

21) Centrifuge samples at 8,000 x g for 1 minute. Place the DNeasy spin column into a clean 2.0 mL collection tube.

22) Add 500 μL of Buffer AW1 to each DNeasy Spin column. Centrifuge DNeasy Spin columns at 8,000 x g for 1 minute.

23) Place the DNeasy spin column into a clean 2.0 mL collection tube.

24) Add another 500 μL of Buffer AW1 to each DNeasy Spin column. Centrifuge all DNeasy Spin columns at 8,000 x g for 1 minute.

25) Place the DNeasy spin column into a clean 2.0 mL collection tube.

26) Add 500 μL of Buffer AW2 to each DNeasy Spin column. Centrifuge all columns at maximum speed for 3 minutes in the microcentrifuge.

27) Place the DNeasy spin column into a clean 2.0 mL collection tube.

28) Centrifuge DNeasy Spin columns for 3 minutes at maximum speed to ensure that all residual ethanol from the wash buffers is removed from the column membranes

29) Transfer each DNeasy Spin column to a new, labeled 1.5 mL microcentrifuge tube

30) Add 125 μL of AE elution buffer to each DNeasy Spin column. Be careful to apply buffer to the center of the filter membrane. Do not touch the tip to the filter. Incubate at room temperature for 2-5 minute.

31) Centrifuge DNeasy Spin columns at 8,000 x g for 2 minutes

32) Transfer the column from the elution tube to a new 2.0 mL centrifuge tube. Reapply the eluate (125 uL) from the elution tube onto the center of the column membrane. Return the spin column to the labeled 1.5 mL elution tube.

33) Incubate columns at room temperature for at least 2 minutes

34) Centrifuge DNeasy Spin columns at 10,000 x g for an additional 2 minutes. The resulting “flow-through” contains the extracted black fly DNA.

35) Store samples at 4°C up to 48 hours until qPCR results are obtained and longer storage at ≤ -20°C.

1. **PCR Amplification for O-vND5 and O-15O Pool screening**
2. Prepare qPCR master mix in a clean area
3. Clean and decontaminate all work surfaces, pipets, centrifuges and other equipment prior to use using with 10% freshly prepared bleach.
4. In the PCR area, place HOT FIREPol Probe qPCR Mix enzyme and primer/probes on cold-block. If frozen, thaw HOT FIREPol Probe qPCR Mix prior to use.
5. Mix the enzyme and primer/probes by inversion 5 times.
6. Briefly centrifuge enzyme and primers/probes and return to ice.
7. Label one 1.5 mL microcentrifuge tube for each qPCR assay mastermix (OvND5, O-150, BAC).
8. Determine the number of reactions (N) to set up per assay based on the number of DNA samples to be tested. Each sample and control is tested in duplicate. It is necessary to make excess reaction mix for the NTC, OvPC, extraction control reactions and for pipetting error.
9. Dispense reagents into each respectively labeled 1.5 mL microcentrifuge tube. After addition of the reagents, mix reaction mixtures by pipetting up and down. ***Do not vortex***.
10. Centrifuge for 5 seconds in microcentrifuge to collect contents at the bottom of the tube, and then place the tube in a cold rack.
11. Set up plates in a 96-well rack or Optical stip tubes with caps.
12. Dispense 9 μL of each master mix into the appropriate wells going across the row as shown


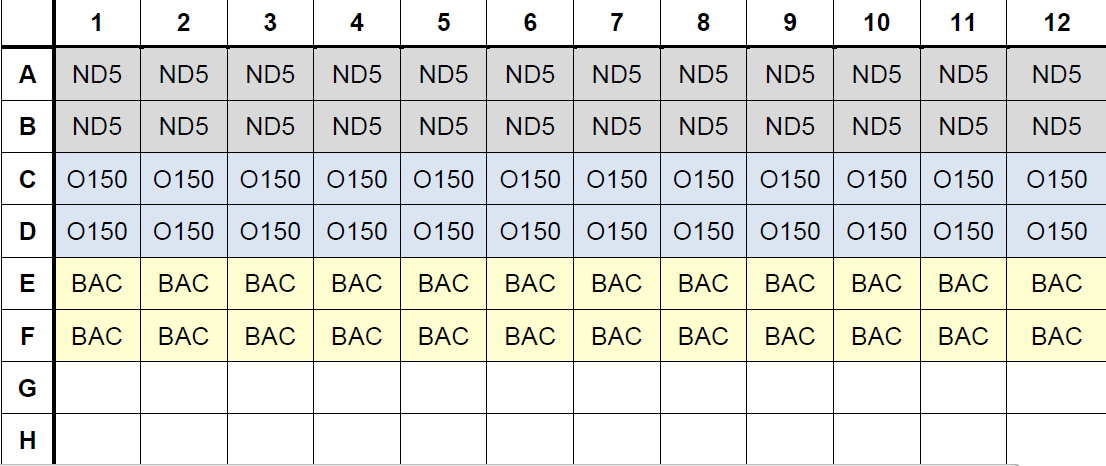


1. Prior to moving to the nucleic acid handling area, prepare the No Template Control (NTC) reactions for column #1 in the assay preparation area.
2. Pipette 1 μL of nuclease-free water into the NTC sample wells (A1~F1).
3. Cover the entire reaction plate with adhesive film (to protect the wells from exposure to particulates in the air) and move the reaction plate to the sample template addition area.
4. In a template addition area, gently vortex DNA sample tubes for approximately 5 seconds.
5. Centrifuge briefly in microcentrifuge.
6. Open sample tubes one at a time away from the plate. Add the sample to its appropriate wells in the plate, and then close the tube before opening the next sample tube with a new tube opener.
7. Samples should be added to wells specific to the assay that is being tested as illustrated below.
8. Pipette sample into specific well carefully and change tips after each addition
9. Cover columns to which the sample has been added to prevent cross contamination and to ensure sample tracking.
10. Change gloves often and when necessary to avoid contamination.
11. After all samples have been added, add 1 μL of the extration control (ExC) sample to the extraction control wells.
12. Pipette 1 μL of OvPC DNA to the sample wells


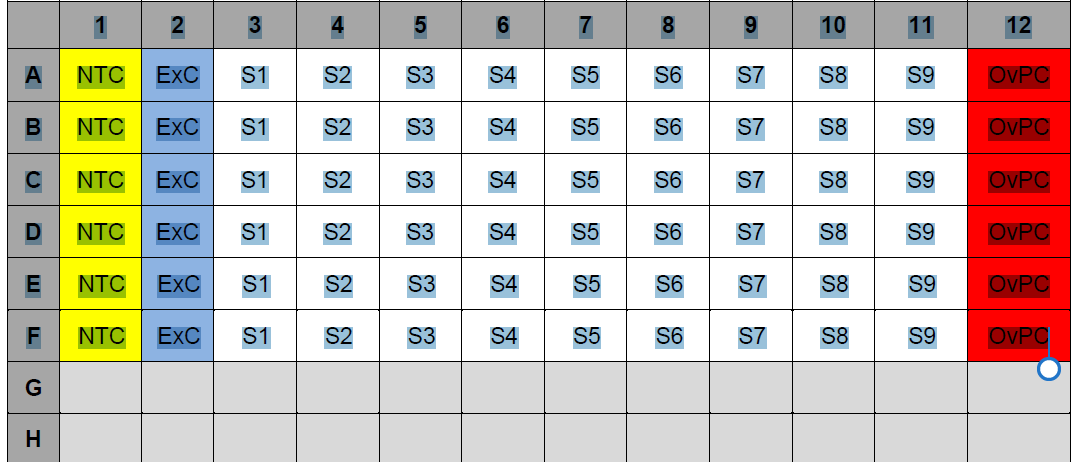


1. Cover the entire reaction plate with adesive film and move the reaction plate to the positive template control handling area.
2. Centrifuge reaction plate or strip tubes for 30 seconds at 500 x g. Inspect plate/tubes to ensure air bubbles are NOT present in any reaction wells
3. Return the DNA samples and reagents to the freezer at ≤ -20oC

**qPCR Reaction Mixtures**

| **Constituent** | **Volume (µl)** |
| --- | --- |
| Template DNA | 1 |
| Combined Primer/Probe Mix | 1 |
| Hot fire pol | 2 |
| ddH_2_O | 6 |
| **Total Volume** | **10** |

**PCR Cycling Condition:**

| **Step** | **Cycles** | **Temp** | **Time** |
| --- | --- | --- | --- |
| Enzyme activation | 1 | 95℃ | 12 min |
| Amplification 40 | | 95℃ | 15 sec |
|  |  | 60℃ | 1 min |

**Data Analysis and Result Interpretation**

1. After completion of the run, save and analyze the data following the instrument manufacturer’s instructions.
2. Analyses is performed separately for each target using a manual threshold setting.
3. The Thresholds is adjusted to fall within exponential phase of the fluorescence curves and above any background signal.
4. Interpretation

***O. volvulus* qPCR Results Interpretation**

| **ND5** | **O-150** | **BAC** | **Result Interpretationa** | **Reporting** |
| --- | --- | --- | --- | --- |
| Ct < 40 | Ct < 40 | ± | *O. volvulus* detected | Report as positive and record Ct values for all samples |
| Undetermined (negative) | Undetermined (negative) | Ct ≤ 35 | *O. volvulus* not detected | Report as negative and record BAC Ct values |
| Ct < 40 | Undetermined (negative) | Ct ≤ 35 | Inconclusive | Repeat. If inconclusive again, report both Ct values and result as inconclusive |
| Undetermined (negative) | Ct < 40 | Ct ≤ 35 | Inconclusive | Repeat. If inconclusive again, report both Ct values and report as inconclusive |
| Undetermined (negative) | Undetermined (negative) | Ct > 35 | Invalid Result | Report as possible inhibition. Retest sample at 1/20 dilution |
